# HPLC-DAD Determination of Iodide in Mineral Waters on Phosphatidylcholine Column

**DOI:** 10.3390/molecules24071243

**Published:** 2019-03-29

**Authors:** Małgorzata Tatarczak-Michalewska, Jolanta Flieger, Justyna Kawka, Wojciech Flieger, Eliza Blicharska

**Affiliations:** Department of Analytical Chemistry, Medical University of Lublin, Chodźki 4A, 20-093 Lublin, Poland; malgorzatatatarczakmichalewska@umlub.pl (M.T.-M.); justyna.kawka@umlub.pl (J.K.); wwoj24@wp.pl (W.F.); bayrena@tlen.pl (E.B.)

**Keywords:** phosphatidylcholine, iodide anions, mineral waters, sustainable chemistry

## Abstract

Iodine is an essential nutrient necessary for the production of thyroid hormones. A valuable source of iodide, which is the bio-available iodine form could be mineral waters offered by different spas. In this work, the method capable of direct determination of iodide in mineral water samples based on IAM liquid chromatography on the phosphatidylcholine column (IAM.PC.DD2 Regis HPLC) with DAD detection without sample pretreatment or any pre-concentration steps is presented. The calibration graph for iodide was linear in the range of 0.5–10.0 mg L^−1^ with a correlation coefficient of 0.9996. The limit of detection was 22.84 ng mL^−1^. The relative recoveries were in the interval of 98.5–100.2% and the repeatability, expressed as a relative standard deviation (RSD) was less than 5%. The RSA (Response Surface Analysis) investigated the effect of the sample concentration and the injection volume. The iodide concentrations in the mineral water samples ranged from 0.58 to 2.88 mg L^−1^. The accuracy of the method was assessed through independent analysis by ICP-MS. Iodide levels measured by these two procedures did not significantly differ. The effects of interfering ions like HCO_3_^−^, Cl^−^, SO_4_^2−^, F^−^, and Br^−^ were also tested. The analysis has shown insignificant differences in the values of the iodide peak area and its height measured in multicomponent mixtures with an error smaller than 5%.

## 1. Introduction

Iodine belongs to an essential micronutrient. For the human body, it is an exogenous element that can only be taken through drinking water or various foods. Iodine is absorbed by the gastrointestinal tract in the form of iodide becoming the key component of the thyroid hormones including thyroxin (T4) and triiodothyronine (T3) [1]. Currently, it is estimated that about 2 billion people in the world are affected by iodine deficiencies, including 30% of which are children [2,3].

Iodine Deficiency Disorders (IDD) cause an endemic goitre, cretinism, and are particularly dangerous for pregnant women since they can lead to fetal brain damage and miscarriage [4,5,6]. Iodine intake for an adult has been estimated to be 1 µg per kg body weight daily [7]. The World Health Organization (WHO) iodine prophylaxis program has recommended obligatory salt iodization of 30 ± 10 mg KI/kg, and additional supplementation for pregnant and lactating women by 150–200 μg iodine per day [2]. The U.S. Institute of Medicine (IOM) has recommended even higher daily allowance (RDA in µg d^−1^) of iodine as follows for adults 150 (RDA), pregnant women: 220 (RDA), and lactating women: 290 (RDA) [8].

Iodine is found mainly as iodide, iodate, and iodine-organic compounds in the environment [9,10]. These forms are converted to one another rapidly. However, iodide is considered to be the most mobile and bioavailable iodine form [10]. Several methods have been described in order to determine the iodine. In most of them, the iodine species were derivatized to iodide, which, subsequently, was determined by a high performance liquid chromatography (HPLC) [11] or to organo-iodine forms further analyzed by a gas chromatography-mass spectrometry (GC-MS) [12]. However, the above methods possess very important limitations such as time-consuming derivatization steps and restricted requirements devoted to the iodine scavenger. The determination of inorganic iodine species is possible by an ion chromatography (IC) with conductivity detection characterizing with poor sensitivity for quantification in some applications. The limitation of IC usability is the presence of chlorides in the matrix, which requires sample pretreatment by the use of an on-guard silver cartridge [13,14].

Other methods recommended for iodine determination go as follows: spectrophotometry [15], titration [16], and the ion-selective electrode [17]. In the case of conventional iodometric titration, the main drawback is false estimation because of interfering oxidizing agents [14]. In turn of electrochemical determination, unsatisfactory precision has been emphasized [18]. Furthermore, the main disadvantages in using electrochemical detection are unvarying pre-treatment steps [19,20,21]. The spectrophotometric method, which is based on the Sandell and Kolthoff reaction [15], is one of the oldest methods utilizing the iodine-catalyzed redox reaction between cerium (IV) and arsenic (III). It has been applied mainly because it does not require expensive instrumentation. However, a catalytic effect of sodium and iron leading to false high values of iodine has been demonstrated [22].

More sophisticated methods such as ion-pair liquid chromatography [23] and Inductively Coupled Plasma Mass Spectrometry, ICP-MS [24], transient isotachophoresis-capillary zone electrophoresis [25], and electroanalysis [26], are recommended as methods for providing reliable and accurate results. However, they have been excluded from routine analysis in many countries due to the high costs. The Dionex application note (Application Note 236) described the use of a silica-based column that incorporates hydrophobic, weak anion-exchange, and ion-exclusion properties (the Acclaim Mixed-Mode WAX-1) to determine iodide in high ionic strength samples such as seawater [27]. In another application note (Application Note 239), an IC system was used with a column designed for the separation of polarizable anions (the Thermo Scientific™ Dionex™ IonPac™ AS20 column) [28]. Conductivity and UV detection showed similar linearity and LOD on the level of 15 µg/L. Linear response to iodide was confirmed between 50–250 µg/L. Determinations of iodide in saline matrixes was accurate with recoveries ranging from 93% to 113%. To determine the concentration of iodide by an HPLC system, the fluorescence detector can also be applied [29,30]. Only one work has described handmade created phosphatidylcholine stationary phases for the chromatographic separation of a few inorganic anions [31]. Obtained stationary phases have been prepared by loading liposomes of dimyristolyphosphatidylcholine (DMPC) onto reversed-phase columns. Currently, Immobilized Artificial Membranes are being prepared commercially by phosphatidylcholine analogues chemically bonded to a propylamino-silica core [32]. As they can efficiently simulate bio-membrane permeation [33,34], these columns are useful in permeability studies or predicting the therapeutic effects of many substances such as (−)-epicatechin conjugates [35], various model drugs [36,37], flavonoids [38], statins [39], and NSAIDs [40].

Taking into account the importance of iodine consumption, it is clear that an analytical method for inorganic iodine determination in different food products that could be easily applied is needed. Therefore, the aim of this work is to develop an analytical method based on IAM-HPLC-DAD capable of quantifying iodide in mineral water samples as a source of iodine supplementation. The proposed methodology is focused on the application of commercially available phosphatidylcholine column (IAM.PC.DD2 Regis HPLC) for the first time, which is able to retain iodide even by the use of water containing NaCl as an eluent, obtain enough capacity necessary for iodide determination in a complex matrix, sufficient sensitivity necessary for trace analysis, selectivity against interfering ions, and using a small sample volume. Obtained results were compared with the reliable ICP-MS method as a reference.

## 2. Results and Discussion

### 2.1. Effect of Experimental Variables

**Figure 1:**
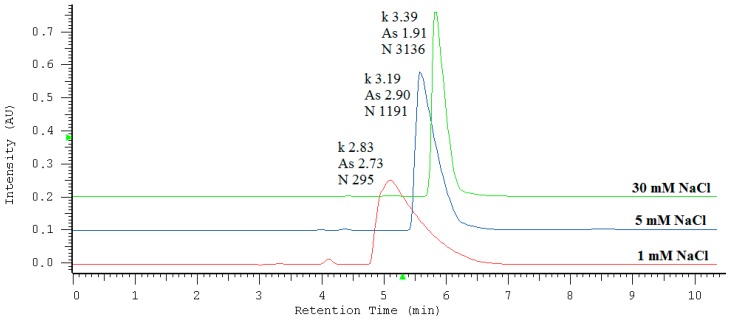
Effect of different concentrations of NaCl in the eluent system on iodide retention, peak symmetry, and efficiency.

To establish the best conditions for the determination of iodide, the high-performance liquid chromatography method was optimized with respect to sodium chloride concentration in the mobile phase. The influence of NaCl on the retention factor (*k*), symmetry (*A*_s_), and efficiency of the peaks (N) may be analyzed on the basis of the data included in Table 1 and the examples of chromatograms obtained under studied conditions (Figure 1). The results show that the retention time increased up to 6 min and then gradually decreased with increasing concentration of NaCl in the mobile phase. A concentration of 30 mM was chosen in subsequent experiments since it enabled the highest efficiency expressed as the theoretical plates number (above 3000 N) and the most beneficial peak’s symmetry (*A*_s_ smaller then 2). It has been previously established that anions are retained on a phospholipid monolayer on the basis of a solvation-dependent mechanism but considering presence of the quaternary ammonium groups (N^+^) on the surface, an anion-exchange process can also contribute to their retention [31]. It is clear that an eluent’s ionic strength influences both retention mechanisms. Therefore, all peak’s parameters undergo improvement with increasing NaCl concentration in the mobile phase.

Chromatographic method optimization was carried out by applying a Response Surface Methodology (RSM). This procedure was applied to obtain optimum chromatographic conditions to avoid volume or concentration overloading, which manifests itself in the form of distorted peak shapes. It allowed the evaluation of the relationship between a set of controlled experimental factors such as injection volume or sample concentration and obtained results expressed as the peak symmetry and the theoretical plate number. The obtained dependency model can be described by the following second-degree polynomial equation.

Z = β_0_ + β_1_C + β_2_C^2^ + V (1)

where Z is a dependent variable, C is the concentration, and V is the injection volume.

**Figure 2:**
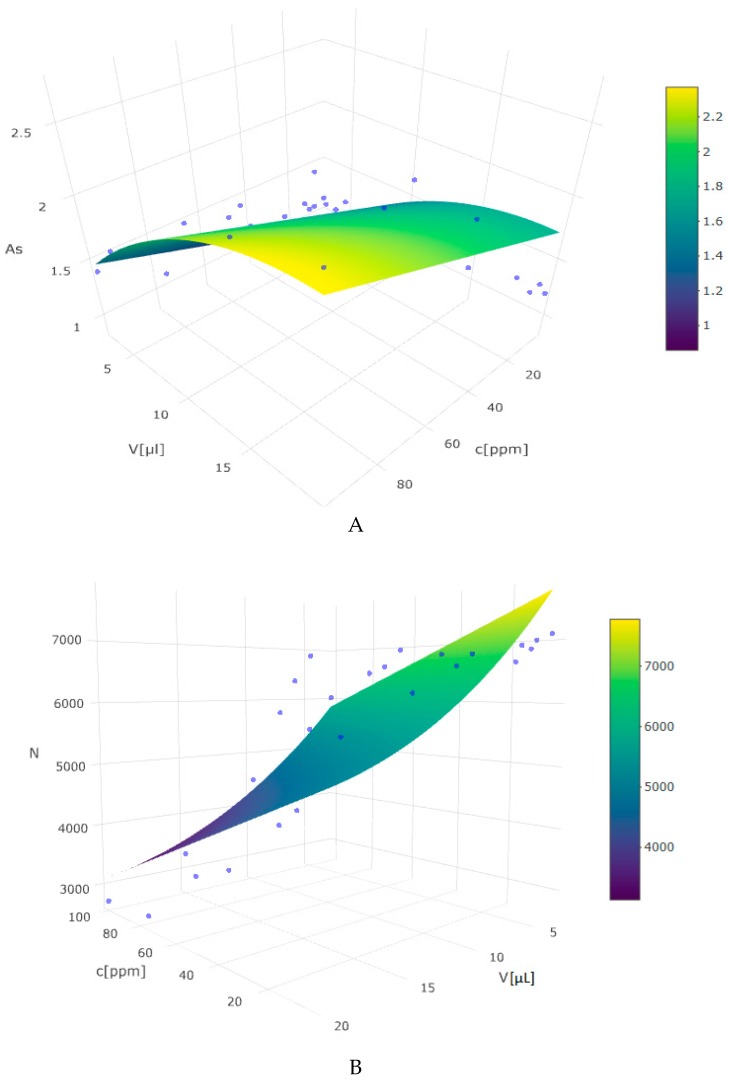
Effect of iodide concentration (C) and injection volume (V) on peak symmetry: *A*s-(**A**), and system efficiency: N-(**B**) by the Response Surface Analysis (RSA).

In terms of matching variables to this model, the relationship between concentration versus the injection volume and the theoretical plate’s number proved to be the best fit. The significance of F statistics in both cases shows that, in each of them, at least one independent variable (concentration or volume) affects the dependent variable (N or *A*s). The response surfaces are shown in Figure 2A,B and Table 2.

In the light of a sequence of designed experiments analyzed by the RSA, the best symmetry of the peak and the system efficiency was obtained by decreasing the sample volume and its concentration. Due to the optimization of operational factors, subsequent quantifications were performed by applying 3 µL of diluted samples. Furthermore, analyzed samples did not require any pre-concentration steps to obtain an optimal response. Such a low sample volume (3 µL) is beneficial when taking into account the possibility of extending the chromatographic column lifetime and avoiding overloading. Other LC-UV applications utilizing the ion-exchange separation mechanism for iodide determination require much bigger sample injection volumes in the range of 25 to 500 µL [11,41,42,43].

### 2.2. Analytical Quality Control

The parameters such as linearity, the limit of detection, and quantification are presented in Table 3.

The applicability of the proposed method was evaluated by analyzing real canal water samples. Since no positive samples were found regarding the content of iodides, the samples were spiked with the analyte at three concentration levels (2, 5, and 10 mg L^−1^). The results are presented in Table 4. Relative recovery values were in the range 98.5–100.2%, which indicates the absence of the matrix effect on the proposed method.

### 2.3. The Mineral Water Sample Analysis

**Figure 3:**
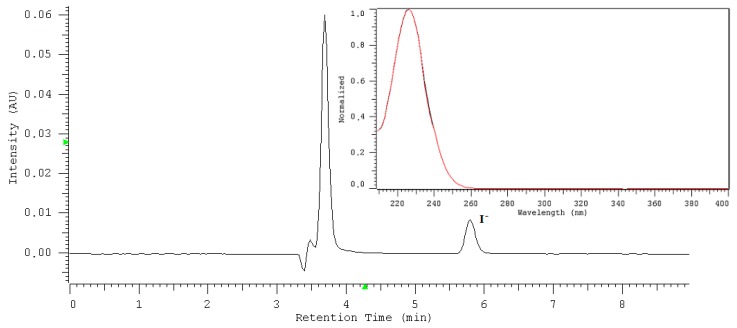
Chromatogram of sample W3. Insert: UV-absorption spectra of iodide in sample W3 recorded in the range of 190 to 400 nm. Conditions: stationary phase: IAM.PC.DD2 Regis HPLC (4.6 × 150 mm, 10 µm, pore size: 300 Å), mobile phase 30 mM NaCl/water, the flow rate 0.5 mL min^−1^, detection 226 nm.

The optimized procedure (IAM-HPLC-DAD) was applied to determine iodide in mineral water samples. Exemplary chromatogram of the investigated water sample with DAD iodide peak identification is presented in Figure 3. Table 5 shows iodide content in six selected natural, mineral waters from Polish resorts with a long tradition in the field of spa medicine. In samples investigated, a single peak occurred at the same retention time, which was measured for a standard iodide. Furthermore, the absorption spectrum measured throughout the chromatographic peak exhibited a maximum at 226 nm, which is characteristic for the iodide. The identity of the iodide was confirmed by retention time and absorption spectrum for the up-and downslope. The coefficient of agreement between the standard spectrum recorded under defined chromatographic conditions and the examined sample was 0.9984.

Iodide was analyzed quantitatively with an external standard curve. The ICP-MS, which is considered to be fast, accurate, robust, and specific, was used as the reference method.

The content of iodides declared by producers differs significantly from the value measured both by HPLC and ICP-MS. In some cases, quantities declared by producers may require re-evaluation to reach true levels. However, the experimental values quantified on the phosphatidylcholine column do not differ from the values measured by the reference ICP-MS method. To investigate the agreement between measurements made by two methods, the relationship between measurements from the IAM-HPLC against those from the reference method was prepared. The linear regression analysis for this dataset possesses the slope close to one and the low value of the intercept, which only differs a little from zero. This indicates excellent statistical compatibility of results obtained by both compared methods.

y (IAM-HPLC) = 0.9607 (± 0.02899)x(ICP-MS) + 0.1379 (± 0.04619)

R^2^ = 0.9856, S_e_ = 0.1056, F = 1098.01

The linear regression might explain the bivariate relationship, which exists between two sets of measurements. Thus, the strength, form, and direction of a relationship can be identified but sometimes this approach is not recommended for comparative analyses [44]. A very useful data visualization tool is the Bland-Altman plot (B & A is known as Tukey Mean-Difference plot). When comparing two sets of measurements for the same variable made by different instruments, it is often required to determine whether the instruments are in agreement or not. The B&A plot is a simple way to evaluate the bias between the mean differences of measurements done by two methods. It is a kind of scatterplot of differences of two paired measurements against the average of these measurements. The average of two measurements is the best way to estimate the true mean. Bland and Altman say that, if the points are between the limits of agreement constructed as a 95% confidence interval for the difference, then these two instruments agree [45,46,47].

**Figure 4:**
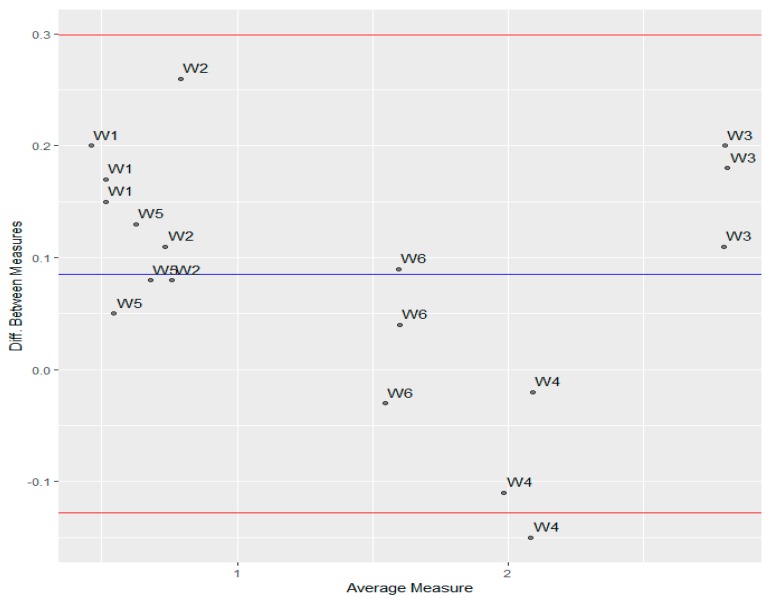
Bland-Altman plot of comparison of two methods (IAM-HLPC and ICP.MS). Blue line represents the mean of differences and red lines are lower and upper bounds of 95% confidence interval for the mean value.

**Figure 5:**
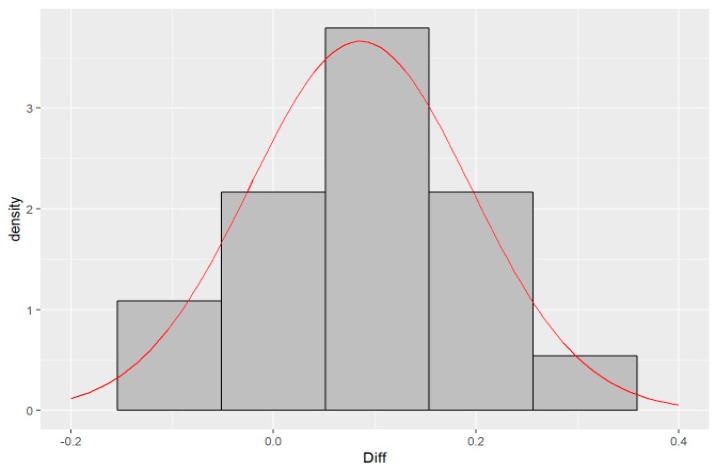
Histogram of differences of measurements with a density of normal distribution.

The gap between the X-axis and the blue line (Figure 4) represents the difference between readings from two measurement instruments. The mean value (0.086) is significantly different from zero (*p* = 0.004). This indicates that, on average, HPLC measurements are higher by about 0.086 units than ICP.MS. Except for one point belonging to sample W4, all observations are between the lower and upper bounds of the 95% confidence interval, which means that these two methods of measuring agree. Furthermore, Bland and Altman suggest checking the assumption of the normal distribution of differences. Figure 5 shows that there is no significant deviation of differences from the normal distribution. This hypothesis was also checked by the Shapiro-Wilk test (*p* = 0.52).

### 2.4. Interferences of Coexisting Ions

The effects of representative potential interfering ionic species in mineral water samples like HCO_3_^−^, Cl^−^, SO_4_^2−^, F^−^, and Br^−^ were also tested. To perform this study, aqueous solutions containing a constant iodide concentration and different coexisting ions were subjected to the developed procedure. Iodide absorbs at 226 nm while the other anions (λ_max_ = 210 nm) remain undetected at this wavelength. Table 6 and Table 7 show the tolerance limits of the interfering ions. The tolerance limit (TL) of coexisting ions was defined as the largest amount, which makes variation less than 5% in the peak height or area.

The presence of bromide provides the highest value of the tolerance limit considering the peak height (4.8%) and its area (3.0), but both of them were still smaller than 5%. Iodide can easily be detected by UV absorbance without interference from other anions. Proposed conditions can be classified as specific for iodide determination since it was either separated from potentially interfering ions, which can be present in mineral water samples at a high concentration or identified by its retention (6 minutes) at absorption maximum (226 nm).

## 3. Experimental

### 3.1. Materials and Sample

Deionized and purified water by ULTRAPURE Millipore Direct-Q 3UV-R (Merck, Darmstadt, Germany) of the resistivity 18.2 MΩ cm was used to prepare all the aqueous solutions. Sodium chloride was sourced from POCH (Gliwice, Poland). A stock standard solution of potassium iodide (100 mg L^−1^) was prepared from potassium iodide (Merck, Darmstadt, Germany). Mineral water samples were purchased from a local market (Lublin, Poland). They were stored at 4 °C in the refrigerator. The working standard solutions were prepared immediately before use.

### 3.2. HPLC Conditions

Chromatographic measurements were made on a LaChrom HPLC Merck Hitachi (E. Merck, Darmstadt, Germany) equipped with a diode array detector (L-7455), pump (L-7100), interface (D-7000), and solvent degasser (L-7612). Chromatographic column IAM.PC.DD2 Regis HPLC (4.6 × 150 mm, 10 µm, pore size: 300 Å) was purchased from Agilent Technologies (Santa Clara, CA, USA). Column temperature was controlled in the range of 15 to 50 °C by the thermostatic column compartment L-7350. Chromatographic data were acquired and processed by D-7000 HSM Software version 3.0 (E. Merck, Darmstadt, Germany). The mobile phases were filtered through a Nylon 66 membrane filter (0.45 µm) Whatman (Maidstone, England) using a filtration apparatus. The eluent was prepared by dissolution inorganic salt (NaCl) in water. The chromatography was carried out at room temperature. Retention data was recorded at a flow-rate of 0.5 mL min^−1^. The detection wavelength was set at 226 nm chosen accordingly with the recorded spectra by the DAD detector ranging from 190 to 400 nm. A sample solution was injected twice ranging from 3 to 20 µL.

### 3.3. ICP-MS

To validate the proposed method, iodide was assayed with the ICP-MS system. Analysis was carried out using an inductively coupled plasma mass spectrometer (ICP-MS), Varian MS-820 (Mulgrave, Victoria, Australia) coupled to a cross-flow nebulizer and a Scott spray chamber. The concentration was determined after diluting the solution with 0.5% (*v*/*v*) tetramethyhylammonium hydroxide (TMAH, Sigma-Aldrich, St. Louis, MO, USA) and adding tellurium as an internal standard. Standard solutions of tellurium with purity of 99.999% were purchased from Ultra Scientific (North Kingstown, RI, USA). Argon gas with a purity of 99.999% was purchased from the Messer Group (Bad Soden, Germany). All reagents were of an analytical grade unless specified otherwise. Deionized water with a resistivity of 18.2 MΩ/cm was obtained from a HLP-20 system (Hydrolab, Straszyn, Poland).

### 3.4. Method Optimization and Validation

The interactions among process variables such as analyte’s concentration and injection volume were determined by the RSA (Response Surface Analysis). The effect of concentration and injection volume was found to range from 5 to 100 ppm (tj. 5, 10, 15, 25, 50, 75, and 100 ppm) and 3–20 µL (3, 5, 10, 15, and 20 µL). The experimental data was developed by using the open source R program [48].

The method validation was carried out according to the guidelines ICH Q2 (R1) [42,49]. The linear range was calculated from a calibration curve made by injecting standards of iodide in triplicate at 10 concentration levels (0.5, 1.0, 2.0, 3.0, 4.0, 6.0, 8.0, 10.0) between 0 and 10 mg L^−1^. The limits of detection (LOD) and quantification (LOQ) correspond to 3 and 10 times the signal-to-noise ratio, respectively. Accuracy of the method was assessed using the determination of iodide recovery. The sample matrix (canal water) was spiked with a known concentration of iodide at three concentration levels of 2, 5, and 10 mg L^−1^ and the percentage of recoveries (R) of the spike standard were calculated as follows:

R (%) = (Cm − Co)/m × 100(2)

where Cm is a value of iodide in a spiked sample, Co is the value of iodide in a sample, and m is the amount of iodide spiked. Specificity of the method was estimated by measuring the response of the investigated analyte (peak height) at a concentration of 4 mg L^−1^ in the presence of the high concentration of the potentially interfering components such as HCO_3_^−^ (1000 mg L^−1^), Cl^−^(1000 mg L^−1^), SO_4_^2−^(100 mg L^−1^), F^−^(10 mg L^−1^), and Br^−^(10 mg L^−1^).

### 3.5. Data Analysis

Multiple regression analysis was performed by using Microsoft Excel 2010. Experimental values were expressed as means ± SD. The Shapiro-Wilk’s W test for detecting a normal distribution of data were performed by the use of Statistica v. 12. The Bland-Altman plot was done in an R-statistical programing environment (R Core Team 2018) [48].

## 4. Conclusions

In the present study, a commercial phosphatidylcholine column IAM.PC.DD2 Regis HPLC was applied for the quantification of iodide in mineral water samples. The obtained results agreed well with ICP-MS used as the reference one, which indicates good performance of both methods for iodide determination. The main advantage of the current work is the simple instrumentation covering HPLC-DAD, which is considered to be a standard in most laboratories. Comparing the current methodology with those previously reported in the literature [50], the method utilizing the HPLC-DAD system with Mixed-Mode WAX-1 column, regarding linearity, sensitivity, precision, and recovery, and the phosphatidylcholine column ensured about two times shorter retention time (current method: 5.8 min, reported method: 10.8 min). In addition, it ensured a ten times smaller LOD value (current method: 22.84 µg L^−1^, reported method: 200 µg L^−1^) and a narrower range of recovery (current method: 98.5–100.2%, reported method: 92.6–108.9%). In conclusion, the presented results prove the usability of the IAM.PC.DD2 Regis HPLC column for iodide determination in aqueous samples.

## Figures and Tables

**Table 1:** Effect of NaCl concentration in the mobile phase in the range of 1 to 30 mM for the KI retention factor (*k*), the peak symmetry factor (*A*_s_), and the theoretical plates number (N). The column was IAM.PC.DD2 Regis HPLC Agilent Technologies, flow rate of the mobile phase was 0.5 mL/min, injected volume 3 μL, and analytical wavelength of 226 nm.

NaCl Concentration [mM]	Retention Factor *k*	Symmetry Factor *A*s *	Theoretical Plates Number N (EUP) **
1	2.83 (± 0.08)	2.73 (± 0.05)	295 (± 97)
5	3.19 (± 0.07)	2.90 (± 0.04)	1191 (± 147)
10	3.57 (± 0.06)	2.97 (± 0.07)	1973 (± 198)
15	3.59 (± 0.10)	2.74 (± 0.08)	2352 (± 116)
20	3.59 (± 0.07)	2.57 (± 0.06)	2794 (± 174)
25	3.50 (± 0.08)	2.32 (± 0.07)	3082 (± 196)
30	3.39 (± 0.06)	1.91 (± 0.06)	3136 (± 177)

All calculations were performed using the HSM program. * The HSM program uses the following equation to calculate asymmetry: *As* = 1/2(1 + B/A), where A and B are evaluated at a 5% peak height of an appropriate peak. ** The following equation is used to calculate the number of theoretical plates according to EUP standards: N = 5.54 (RT/W_1/2_), where RT is the actual retention time of the appropriate peak, W equals the peak width obtained by drawing tangents to each side of the peak and calculating the distance between the two points where the tangents meet a line that runs parallel to the baseline at half peak-height.

**Table 2:** Second-degree polynomial model parameters.

Parameters	*A*s	N
C	0.021 (0.005)	−49.876 (11.734)
I(C2)	−0.0001 (0.00004)	0.204 (0.112)
V	0.037 (0.006)	−113.931 (14.506)
constant	0.646 (0.100)	8360.409 (257.425)
observations	35	35
R^2^	0.812	0.853
Adjusted R^2^	0.794	0.839
Residual Std Error	0.209 (df = 31)	538.961 (df = 31)
F statistic	44.771 (df = 3;31)	60.201 (df = 3;31)

**Table 3:** The linear regression parameters obtained for the calibration curves of iodide ions.

LOD * [mg L^−1^]	LOQ * [mg L^−1^]	Linear Range * [mg L^−1^]	Regression Equation ** (y = ax + b)				
			a ± S_a_	b ± S_b_	r	S_e_	F
0.02284	0.06852	0.5–10.0	16156.1 (± 175.33)	−2264.6 (± 902.5)	0.9996	1361.5	8491.4

* Number of calibration points: 8. ** S_a_: the standard deviation of the slope, S_b_: the standard deviation of the intercept, S_e_: the standard error of estimate, F—Fisher F Statistic.

**Table 4:** The percentage of recoveries (R) of real canal water samples spiked with the analyte.

Sample	Added [mg L^−1^]	Measured Mean ± SD (n = 3) [mg L^−1^]	Percentage of Recovery	RSD %
Canal water	0	0	-	-
	2	1.97 (± 0.07)	98.5	3.55
	5	4.96 (± 0.22)	99.2	4.43
	10	10.02 (± 0.46)	100.2	4.59

**Table 5:** Iodide content in selected mineral waters (n = 3 each) analyzed by HPLC-DAD on IAM.PC.DD2 Regis HPLC Agilent Technologies and ICP-MS.

Sample	Contents Declared [mg L^−1^]	IAM.PC.DD2 Regis HPLC Agilent Technologies [mg L^−1^] Mean ± SD	Relative Bias * (%)	ICP-MS [mg L^−1^] Mean ± SD	Relative Bias ** (%)
1	0.52	0.58 (± 0.02)	11.5	0.41 (± 0.04)	−21.1
2	2.49	0.84 (± 0.07)	−66.2	0.69 (± 0.03)	−72.2
3	2.50	2.88 (± 0.03)	15.2	2.72 (± 0.02)	8.8
4	2.36	2.01 (± 0.07)	−14.8	2.10 (± 0.06)	−11.0
5	0.90	0.66 (± 0.08)	−26.6	0.57 (± 0.06)	−36.6
6	4.43	1.60 (± 0.06)	−63.8	1.56 (± 0.02)	−64.8

* Relative bias (%) = (value from HPLC − Contents declared) × 100/Contents declared. ** Relative bias (%) = (value from ICP-MS − Contents declared) × 100/Contents declared.

**Table 6:** The tolerance limits of the interfering ions in the height of the iodide peak.

Statistical Evaluation of the Iodide Peak Height [I^−^] = 4 ppm	H_2_O	HCO_3_^−^ [1000 ppm]	Cl^−^ [1000 ppm]	SO_4_^2−^ [100 ppm]	F^−^ [10 ppm]	Br^−^ [10 ppm]
Average peak height	6188.50	6209.67	6263.67	6138.67	6140.33	5890.33
SD	254.26	100.01	59.70	274.97	109.70	157.02
CV	4.11	1.61	0.95	4.48	1.79	2.67
Tolerance limit [%]	-	0.3	1.2	0.8	0.7	4.8

**Table 7:** The influence of selected anions on the surface area of iodine peaks in the standard solutions.

Statistical Evaluation of the Iodide Peak Area [I−] = 2 ppm	H_2_O	HCO_3_^−^ [1000 ppm]	Cl^−^ [1000 ppm]	SO_4_^2−^ [100 ppm]	F^−^ [10 ppm]	Br^−^ [10 ppm]
Average peak area	232,474.7	231,092.7	233,938.9	230,291.6	230,719.1	225,425.5
SD	3185.09	3122.82	3182.58	2255.27	1715.77	4106.21
CV	1.37	1.35	1.36	0.98	0.74	1.82
Tolerance limit [%]		0.6	0.6	0.9	0.8	3.0
